# *In vitro* Susceptibility and Evaluation of Techniques for Understanding the Mode of Action of a Promising Non-antibiotic Citrus Fruit Extract Against Several Pathogens

**DOI:** 10.3389/fmicb.2019.00884

**Published:** 2019-04-24

**Authors:** Pedro J. G. de Nova, Ana Carvajal, Miguel Prieto, Pedro Rubio

**Affiliations:** ^1^Department of Animal Health, Faculty of Veterinary, Universidad de León, León, Spain; ^2^Institute of Food Science and Technology, Universidad de León, León, Spain; ^3^Department of Food Hygiene and Technology, Faculty of Veterinary, Universidad de León, León, Spain

**Keywords:** susceptibility, antimicrobial, antibacterial, phytobiotics, plant extracts, BIOCITRO, alternative to antibiotics, mode of action

## Abstract

The screening for alternatives to antibiotics is an urgent need for the pharmaceutical industry. One of these alternatives seems to be the citrus fruit extracts, which are showing a significant antibacterial activity against Gram-negative and Gram-positive bacteria. One of these citrus extracts, named BIOCITRO^®^, is assessed in this study to elucidate its bacteriostatic and bactericidal effect and its mode of action on the important pathogens *Campylobacter coli*, *C. jejuni*, *Escherichia coli*, *Salmonella enterica* ssp. *enterica*, *Clostridium difficile*, *C. perfringens*, and *Staphylococcus aureus*. For most of the strains tested of these bacteria the product was bactericidal as well as bacteriostatic at the same concentration, and the minimum bactericidal concentrations ranged from 16 to 256 μg/mL. Regarding the mode of action, important changes in the permeability, structure, composition and morphology of the bacterial envelope were evidenced using flow cytometry, Fourier transform infrared spectroscopy and scanning electron microscopy. The main effect of the product was found over carbohydrates and polysaccharides, inducing the release of microvesicles by the cells in addition to other specific effects.

During the study, the techniques used were evaluated to clarify their contribution to the knowledge of the mode of action of the product. The survival test elucidated whether the modifications displayed using other techniques affected the viability of the cells or on the contrary, the cells remained viable even with evident changes in their structure, composition or morphology. Flow cytometry showed that for some strains the proportion of cells detected with altered membrane permeability were higher than the number of non-viable cells, and therefore the damage did not affect the viability of some cells. On the contrary, some cells observed using scanning electron microscopy with no apparent damage, were demonstrated non-viable using the survival test, making this technique indispensable in studies of the mode of action of antimicrobials to make a correct interpretation of the data from other techniques.

## Introduction

The increase in the resistance of microorganisms to antibiotics makes it necessary to explore alternative products for the control and prevention of diseases. Some plant extracts have been shown as effective antimicrobials by many *in vitro* and even some *in vivo* experiments (Rong et al., 2016; Delić et al., 2018). Besides, beneficial changes in the microbiota of the gastrointestinal tract have been described when they are used as feed additives (Manzanilla et al., 2004; Castillo et al., 2006). Of these plant extracts, citrus fruit extracts are showing an acceptable antibacterial activity against a broad range of Gram-negative and Gram-positive bacteria. Therefore, they are recommended as feed additives to improve animal health (Alvarez-Ordóñez et al., 2013), to control food-borne pathogens (Iturriaga et al., 2012; Vardaka et al., 2016; Tsiraki et al., 2018), against helminths (Abdelqader et al., 2012), as surface cleaners (Cormier et al., 2013) or even against insect pests on plants (Hollingsworth, 2005). BIOCITRO^®^^1^ is a commercialized citrus fruit extract obtained from organic cultures of grapefruit (*Citrus paradisi*), tangerine (*Citrus reticulata blanco*), bergamot (*Citrus aurantium* ssp. *bergamia*), and sweet orange (*Citrus sinensis*). It was previously described as a powerful antibacterial agent against *Brachyspira hyodysenteriae* (de Nova et al., 2017) and some food-borne pathogens (Bevilacqua et al., 2010).

As BIOCITRO^®^ is recommended as feed or drink supplement to control digestive disorders in livestock, the use of representative species of the Gram-negative and Gram-positive groups of etiological agents of these diseases is necessary to achieve a correct knowledge of the global activity of the product. It must also be taking into account that some of the species that cause livestock digestive diseases are zoonotic and therefore of great interest for the human health. In this way, the World Health Organization^2^ considers *Campylobacter*, *E. coli*, and *Salmonella*, three of the most common causes of diarrhea in livestock, as important zoonotic agents. In addition, *E. coli* and methicillin-resistant *Staphylococcus aureus* (MRSA) are used as indicator bacteria in the surveillance of antimicrobial resistance by the European Food Safety Authority (EFSA and ECDC, 2018) making these bacteria very important in the assessment of alternatives to antibiotics. Another important genus involved in digestive disorders is *Clostridium* which includes *C. difficile*, one of the main causative agents of nosocomial diseases.

In this study, the *in vitro* antimicrobial activity of BIOCITRO^®^ was evaluated in a set of 47 strains belonging to five genera of representative Gram-positive and Gram-negative pathogens, in order to establish the bacteriostatic and bactericidal concentrations of the citrus extract. Moreover, two strains representing the lowest and highest susceptibility of each genera were assessed to understand the mode of action of the product over the different bacterial groups using flow cytometry (FC), fourier transform infrared (FTIR) spectroscopy and scanning electron microscopy (SEM). These techniques are capable of showing the molecular and structural changes which take place in the cells and combined with the study of the viability of the cell (survival test) can clarify the significance of the modifications displayed using these techniques in the viability of the cells.

## Materials and Methods

### Bacterial Strains and Growth Conditions

A set of 30 Gram-negative and 17 Gram-positive strains belonging to five genera of bacteria from the Infectious Diseases Unit Collection of the University of León in Spain were selected for the assessment (Table 1). Tryptic soy agar (Scharlau) and Mueller-Hinton broth (Cultimed) as solid and liquid media, respectively, were used to grow *E. coli*, *Salmonella enterica* ssp. *enterica*, and *S. aureus*; Columbia agar with sheep blood (Oxoid) and brain heart infusion broth (Merck) supplemented with 0.4% yeast extract for the growth of *Campylobacter* spp.; and fastidious anaerobe agar (Amersham) and brain heart infusion broth for the culture of *Clostridium* spp. Bacteria were grown in both solid and liquid media for 24 h at 37°C with the exceptions of *Campylobacter* spp. and *Clostridium* spp. that were maintained for 48 h. Liquid cultures were grown on an orbital shaker at 60 rpm. Microaerophilic conditions for the culture of *Campylobacter* spp. were achieved using a jar with a pack of CampyGenTM (Oxoid), whereas anaerobic bacteria were incubated on solid medium in an anaerobic chamber (80% N_2_, 10% H_2_, and 10% CO_2_), or in a jar with a pack of AnaeroGenTM (Oxoid) when liquid medium was used. The bacteria were always resuscitated on solid medium plates to check purity before each assay and all the experiments described below were carried out in triplicate.

**Table 1:** Bacterial strains used in the study and their susceptibility to the citrus fruit extract (μg/mL).

					MIC		MBC	
	
Id.	Source	Strain	MIC	MBC	50	90	50	90
*Campylobacter coli*					32	32	32	32
CC8766	IDUC	*C. coli*	32	32				
CC10131	IDUC	*C. coli*	32	32				
CC10604	IDUC	*C. coli*	32	32				
*Campylobacter jejuni*					32	32	32	32
DSMZ4688T	DSMZ	*C. jejuni*	32	32				
CJ34	IDUC	*C. jejuni*	32	32				
CJ60	IDUC	*C. jejuni*	32	32				
CJ6937	IDUC	*C. jejuni*	32	32				
CJ7672	IDUC	*C. jejuni*	32	32				
CJ7674	IDUC	*C. jejuni*	32	32				
CJ10647	IDUC	*C. jejuni*	32	32				
*Escherichia coli* (hemolytic strain)					128	128	128	128
EC58	IDUC	*E. coli*	128	128				
EC59	IDUC	*E. coli*	64	64				
EC60	IDUC	*E. coli*	128	128				
EC61	IDUC	*E. coli*	64	128				
EC62	IDUC	*E. coli*	128	128				
EC63	IDUC	*E. coli*	128	128				
EC64	IDUC	*E. coli*	128	128				
EC65	IDUC	*E. coli*	128	128				
EC66	IDUC	*E. coli*	64	64				
EC67	IDUC	*E. coli*	128	128				
*Salmonella enterica* ssp. *enterica* (serovar)					128	128	128	256
CECT443	CECT	*Salmonella* Typhimurium	64	128				
CECT883	CECT	*Salmonella* Typhimurium	128	128				
CECT4300	CECT	*Salmonella* Enteritidis	64	128				
CECT700	CECT	*Salmonella* Infantis	128	256				
CECT915	CECT	*Salmonella* Choleraesuis	128	128				
SP11	IDUC	*Salmonella* Typhimurium DT104	128	128				
SP28	IDUC	*Salmonella* London	128	128				
SP36	IDUC	*Salmonella* Rissen	128	128				
SP58	IDUC	*Salmonella* Typhimurium DT104	128	128				
SP62	IDUC	*Salmonella* Derby	64	128				
*Clostridium difficile*					32	32	64	64
CD1	IDUC	*C. difficile*	32	64				
CD2	IDUC	*C. difficile*	16	16				
CD4	IDUC	*C. difficile*	16	64				
CD5	IDUC	*C. difficile*	32	64				
CD38	IDUC	*C. difficile*	32	64				
*Clostridium perfringens*					16	16	16	16
CP3	IDUC	*C. perfringens*	16	16				
CP34	IDUC	*C. perfringens*	16	16				
CP52	IDUC	*C. perfringens*	16	16				
CP89	IDUC	*C. perfringens*	16	16				
CP99	IDUC	*C. perfringens*	16	16				
*Staphylococcus aureus*					32	64	32	64
MRSA1	IDUC	*S. aureus*	64	64				
MRSA2	IDUC	*S. aureus*	64	64				
MRSA3	IDUC	*S. aureus*	32	32				
MRSA4	IDUC	*S. aureus*	32	32				
MRSA5	IDUC	*S. aureus*	16	32				
CECT4459	CECT	*S. aureus*	16	16				
CECT4465	CECT	*S. aureus*	16	32				


*CECT, Spanish type culture collection; DSMZ, German collection of microorganisms and cell Cultures; IDUC, Infectious Diseases Unit Collection of the University of León-Spain; MIC, minimum inhibitory concentration; MBC, minimum bactericidal concentration; MRSA, Methicillin-resistant Staphylococcus aureus.*

### Minimum Inhibitory Concentration (MIC) and Minimum Bactericidal Concentration (MBC)

The broth microdilution method was used to assess the concentrations of the product which inhibited the growth MIC and those which killed the bacteria MBC. Briefly, two-fold serial dilutions of BIOCITRO^®^ were prepared in 96 well tissue culture plates, in a final volume of 100 μL of the liquid medium previously described for each genus. Bacterial cells from a fresh culture plate were suspended in 2 mL of 0.85% NaCl to achieve an equivalent turbidity of 0.5 McFarland standard solution (approximately 1.5 × 10^8^ colony-forming units (CFU)/mL). The suspension was diluted (6.67 μL/mL) in the appropriate liquid medium to reach a final concentration of approximately 1 × 10^6^ CFU/mL (CLSI, 2012). Next, 100 μL of the bacteria suspension were added in each well, so that the final inoculum concentration was 5 × 10^5^ CFU/mL in each well. Negative and positive controls that only contained the liquid medium without or with inoculum, respectively, were included. The MIC was obtained as the minimum concentration that inhibited visual bacterial growth after incubation under the conditions previously described for the liquid culture of each genus. In addition, the MBC was considered as the minimum concentration in which no growth was visually observed after plating, on the appropriate solid medium, 20 μL of the culture from each well where there was no apparent growth after the incubation in the tissue culture plates, detecting 99.99% of eradication of the initial inoculum of 5 × 10^5^ CFU/mL. The cultures of the wells with positive growth with the higher concentration of the extract were included as positive controls. When the results differed between the three replicated experiments, the value of the two matching replicas was taken. In order to compare the homogeneity in the behavior of the strains of the same species, the values of MIC_50_, MBC_50_, MIC_90_, and MBC_90_ were calculated as the concentration that inhibited MIC or killed MBC 50 or 90% of the isolates tested for each bacterial species.

In order to perform the studies described below, two representative strains of each genus corresponding to the highest and lowest values of the MBC within the genus (MBCg) were selected. Stationary phase cultures grown in the appropriate liquid medium as previously described were divided in five aliquots. Four of them were exposed for 90 min at room temperature to four different concentrations of BIOCITRO^®^: half the lowest MBCg (½LMBCg), the lowest MBCg (LMBCg), the highest MBCg (HMBCg) and twice the highest MBCg (2HMBCg). On the other hand, the remaining aliquot, hereafter referred as the “control,” was maintained for 90 min at room temperature without the product and subsequently subjected to the same protocols used for the cells exposed to the product, to know the damages caused by the handling of samples in each technique. For *S. enterica* ssp. *enterica* serovar Typhimurium two strains were included to compare the effect of BIOCITRO^®^ on the special phagotype DT104 (*S.* Typhimurium SP11) which is characterized by multiresistance to antibiotics.

### Membrane Integrity Test Using Flow Cytometry

Alterations of the membrane permeability to propidium iodide (PI) (Molecular Probes, Invitrogen, Life Technologies) by BIOCITRO^®^ were detected using FC in the two selected strains of each genus. The stationary phase cultures were adjusted to a final concentration of ∼10^8^ cells/mL with phosphate-buffered saline (PBS, pH 7.4) and PI was added to a final concentration of 0.1% (v/v) for ten min at room temperature in the dark (de Nova et al., 2017). Cells with and without PI were detected in a CyAn-adp flow cytometer (Beckman Coulter) with a laser excitation of 488 nm, the detection at red fluorescence (613/20 nm) and the configuration of forward scatter (FS) and side scatter (SS). Cell debris and aggregates were excluded by gating in a FS vs. SS dot plot (Supplementary Figure S1) and events were calculated from a fluorescence histogram produced from a 10000 particle count in the gate (Supplementary Figures S2–S6). Data were analyzed with Summit version 3.1 software (Cytomation) to obtain the amount of stained and non-stained cells. The averages and standard deviations of the percentage of stained cells detected in the three replicates for each treatment and strain were represented with Excel 2013 (Microsoft). For each strain, the percentages of stained cells of the three replicates of each treatment were statistically compared only against the percentages of the three replicates of its control using SPSS Statistics v24 (IBM) and the ANOVA at α = 0.05 in order to show the lowest concentration at which the product started to have significant effects with regard to the control.

### Changes in the Medium Infrared (MIR) Spectra

Fourier transform infrared spectroscopy was used to evaluate changes in the cellular structure and composition as was previously described (Alvarez-Ordóñez et al., 2013). Stationary phase cultures, after the treatment previously mentioned, were centrifuged at 11000 *g* for 3 min at 4°C. The pellet was suspended in 15 μL of PBS and desiccated on a ZnSe window (Maradona, 1996; Mouwen et al., 2005). The MIR spectra were obtained averaging 20 scans in the range from 3500 to 700 cm^-1^ with an interval of 1 cm^-1^ and a spectral resolution of 4 cm^-1^ in a FTIR spectroscope (Perkin-Elmer 2000 FTIR). A set of 2800 points of the digitized spectra were saved and mathematically processed. To avoid methodological variations and maximize the differences between spectra, normalization of the spectra (0 setting absorption at 1800 cm^-1^; 1 setting at maximal absorption around 1650 cm^-1^), smoothing and second derivative (Savitzky-Golay algorithm) were performed using an application developed for the Perkin-Elmer environment. The five spectral windows previously defined from the whole spectrum (Naumann et al., 1991; Alvarez-Ordóñez and Prieto, 2012) were considered for calculation purposes: the windows w_1_ (3000–2800 cm^-1^), w_2_ (1800–1500 cm^-1^), w_3_ (1500–1200 cm^-1^), w_4_ (1200–900 cm^-1^), and w_5_ (900–700 cm^-1^). Reproducibility of the three replicates of each strain (within-group variability) was calculated using individual Pearson’s product-moment-correlation coefficient as *D* or differentiation index (Alvarez-Ordóñez and Prieto, 2012), and averaging the *D*-values for the replicates 1–2, 2–3, and 1–3. Window w_4_ was selected because it obtained the lowest *D*-values, which means highest reproducibility between replicates, and also showed the highest differences of the five windows when compared the control with the highest concentration used. After normalization, smoothing and second derivative transformation, data from the w_4_ window were collected in ASCII format to graphically represent the averages of the replicas and to display the differences between each experiment by factor analysis (FA) using Statistica for Windows v. 7.0 software (Statsoft Inc.).

### Scanning Electron Microscopy (SEM)

Morphological and physical changes in four of the most susceptible strains of two representative genera in the study (two Gram-negative and two Gram-positive strains) were qualitatively studied using SEM. Only the exposures to the two highest concentrations of BIOCITRO^®^ (HMBCg and 2HMBCg) and the control (0 μg/mL) were observed. After the treatment the bacterial cells were centrifuged at 2400 *g* for 6 min, resuspended in 2.5% glutaraldehyde in PBS and maintained at 4°C for 24 h, as was previously described (de Nova et al., 2017; Kuo, 2007). The samples were then washed three times with PBS and post-fixed with 1% osmium tetroxide in PBS for 45 min in the dark. Afterward, they were washed again three times with PBS and filtered on polycarbonate membrane (Isopore, 0.2 μm, Millipore), dehydrated in graded ethanol series (30%, 50%, 70%, 90%, 3 × 96%, and 3 × 100%, each for 10 min) and dried using the critical point method (CPD 030, Balzers). Finally, they were mounted on aluminum stubs with conducting carbon ribbon and coated using sputter coater (SCD 004, Balzers). For the observation of the samples and capture of the digital images a JEOL 6480LW scanning electron microscope was used operating at 20 kV.

### Survival Test

The viability of the cells was assessed following an adaptation of a previously described method (Alvarez-Ordóñez et al., 2013). After the treatment of the stationary phase cultures with and without the product for 90 min at room temperature, each of the combinations of strain-BIOCITRO^®^ and the control were diluted in ten-fold series in PBS. Suitable dilutions containing between 25 and 250 CFU per 100 μL were plated on solid media in duplicate for each assay of the three replicate experiments. After growing, the viable cells counted from the two plates were averaged and the surviving populations estimated for each experiment. Averages and standard deviations of the three replicates of each experiment in logarithmic scale were represented with Excel 2013 (Microsoft). SPSS Statistics v24 (IBM) and the ANOVA at α = 0.05 were used to calculate the lowest concentration of the product in which significant differences with regard to the control occurred.

## Results

### MIC and MBC

Most of the independent replicates performed for each strain to evaluate the MIC and MBC showed the same value, whereas variable replicates differed no more than one dilution step. The values ranged from 16 to 128 μg/mL for the MIC and 16 to 256 μg/mL for the MBC (Table 1). In 36 of the 47 strains (76.6%) the MIC was the same as the MBC, ten strains showed their MBC as being twice the MIC (21.3%) and only the MBC of one strain of *C. difficile* differed two dilution steps of its MIC (2.1%). There were no differences between the MIC_50_, MIC_90_, MBC_50_, and MBC_90_ of the genus *Campylobacter* (32 μg/mL), either for *E. coli* (128 μg/mL) or *C. perfringens* (16 μg/mL). On the other hand, the MBC_90_ of *S. enterica* ssp. *enterica* (256 μg/mL) was twice its MIC_50_, MIC_90_, and MBC_50_; the MBC_50_ and MBC_90_ of *C. difficile* presented identical concentration (64 μg/mL) which were twice its MIC_50_ and MIC_90_; and the MBC_90_ of *S. aureus* coincided with its MIC_90_ (64 μg/mL) and was twice the concentration of the MBC_50_ and MIC_50_ which were both the same. Thus, the genera with the highest and lowest susceptibility of the study were *C. perfringens* with MBC_90_ of 16 μg/mL and *S. enterica* ssp. *enterica* with MBC_90_ of 256 μg/mL though its MIC_90_ was 128 μg/mL. Furthermore, both species of the genus *Campylobacter* were inhibited and killed at the same concentration of 32 μg/mL showing the most homogeneous values of MIC and MBC of all genera.

After the evidence of the antimicrobial activity of the product, the study of the mechanism of action by which such activity occurs was carried out. We performed preliminary experiments with cells of the stationary phase of growth treated with and without the product at 60, 90, and 120 min at room temperature (data not shown) and the results were very similar, suggesting a saturation of the effects or a very quick depletion of the product. Thus, the next experiments to evaluate the mode of action of the BIOCITRO^®^ were performed using a single incubation time at 90 min at room temperature, knowing that the main effects had already been achieved by this time.

### Membrane Integrity Test Using FC

The effects on the cellular membrane permeability after the exposition to the citrus extract were evaluated using FC thanks to the properties of PI which binds to the DNA and produces red fluorescence. The PI is not capable of crossing the cellular membrane unless it is damaged, therefore, only cells with sufficient alteration of their membrane permeability can contain PI attached to their DNA and FC can discriminate between cells with PI (with altered membrane) and without PI (with undamaged membrane).

**FIGURE 1:**
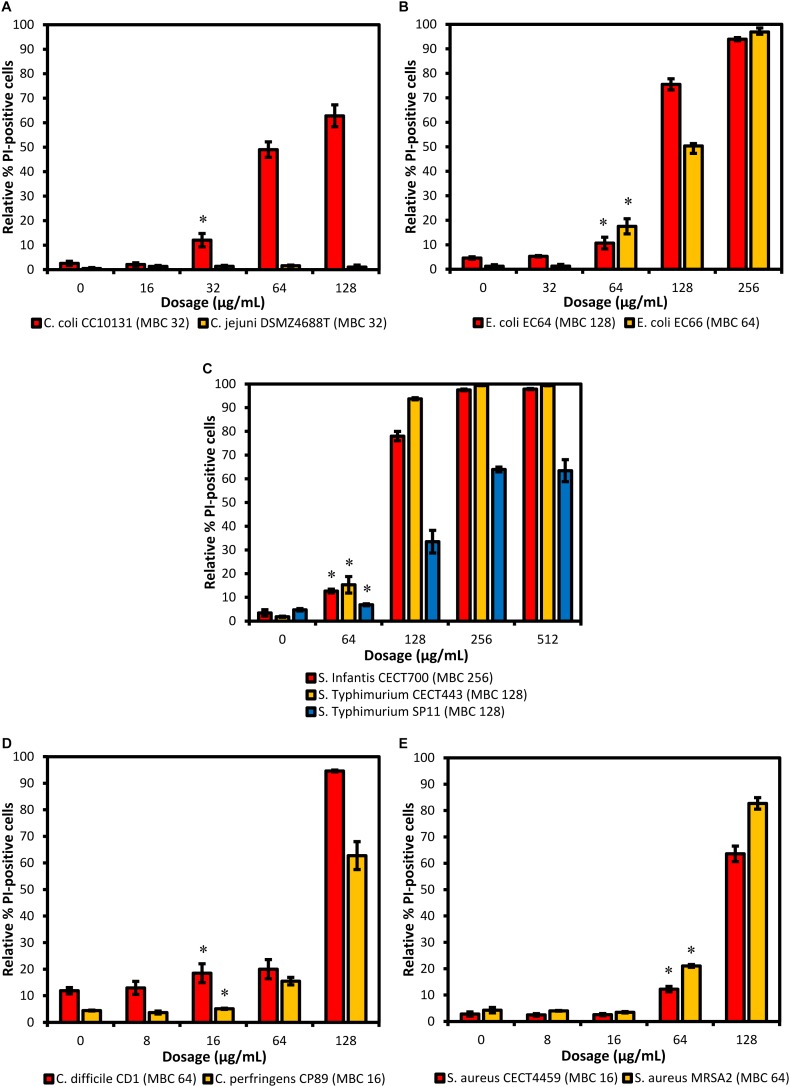
Proportion of cells stained with PI (altered membrane permeability) detected using FC after 90 min of exposure to different concentrations of BIOCITRO^®^ (half the lowest, the lowest, the highest, and twice the highest MBC of each genus). Data show the averages of the three independent replicate experiments for each strain grouped by genus: **(A)** *Campylobacter*, **(B)** *E. coli*, **(C)** *S. enterica* ssp. *enterica*, **(D)** *Clostridium*, and **(E)** *S. aureus*. The differences between the average of damaged cells of each treatment with regard to the control (without product) were significant for the concentrations marked with ^∗^ and higher.

The proportion of stained cells with PI after their exposition to the different concentrations of the product are shown in Figure 1. The strain of *C. jejuni* DSMZ4688T showed no detectable damage with any of the concentrations tested and was not evaluated using FC. Moreover, there was mild damage in the controls of all the strains, averaging 4.45% of stained cells and ranging from 1.25% for *E. coli* EC66 to 11.88% for *C. difficile* CD1. An important increase in the effect related with the concentration was generally observed with the exception of the highest concentration of *S.* Typhimurium SP11. The lowest concentration of BIOCITRO^®^ at which a significant increase of cells with PI was detected with regard to the control was 16 μg/mL for the genus *Clostridium* (*P* = 0.038 for *C. difficile* CD1 and *P* = 0.013 for *C. perfringens* CP89), 32 μg/mL for *C. coli* CC10131 (*P* = 0.004) and 64 μg/mL for *E. coli* (*P* = 0.012 for *E. coli* EC64 and *P* = 0.001 for *E. coli* EC66), *S. enterica* ssp. *enterica* (*P* ≤ 0.001 for *S.* Infantis CECT700, *P* = 0.003 for *S.* Typhimurium CECT443 and *P* = 0.004 for *S.* Typhimurium SP11) and *S. aureus* (*P* ≤ 0.001 for the two strains). The maximum values of damaged cells were achieved at the concentration of 2HMBCg, with the exception of *S. enterica* ssp. *enterica* which reached the maximum detectable damage at the concentration of the HMBCg even for the phagotype DT104. These maximum values of detectable cells with PI were about 63% of the cells for *C. coli* CC10131, *S.* Typhimurium SP11, *C. perfringens* CP89 and *S. aureus* MRSA2; 82.72% for *S. aureus* CECT4459; approximately 94% for *E. coli* EC64 and *C. difficile* CD1; and exceeded 96% in *E. coli* EC66, *S.* Infantis CECT700 and *S.* Typhimurium CECT443 (Figure 1).

### Changes in the MIR Spectra

**FIGURE 2:**
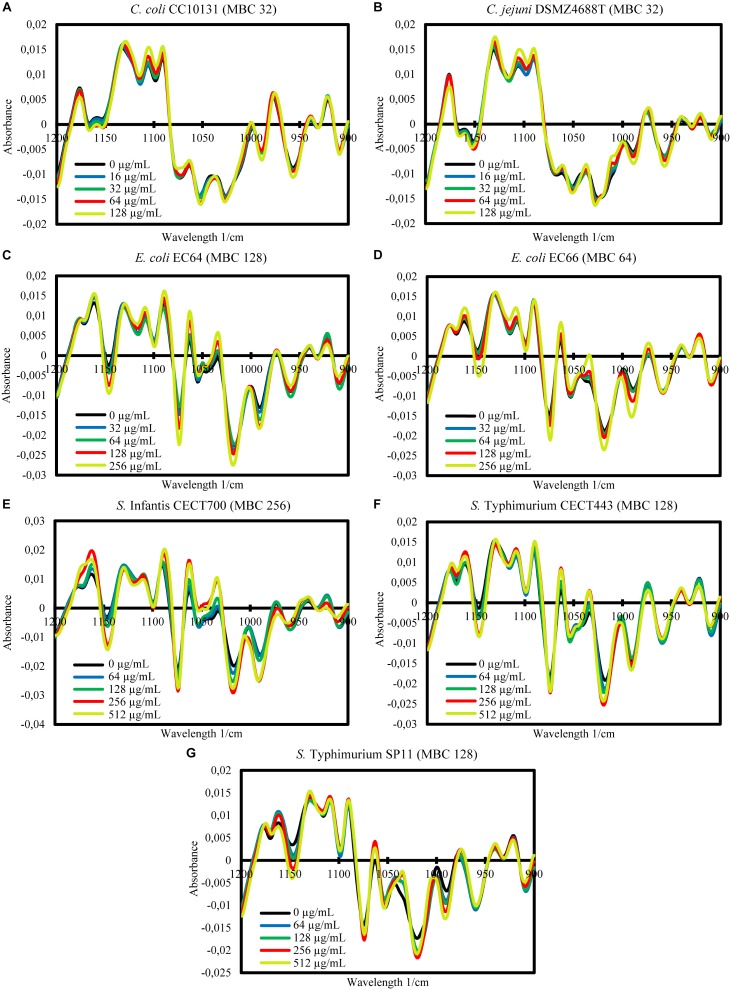
Second-derivative transformation of the FTIR absorbance spectra of the w_4_ window of the selected Gram-negative strains of *Campylobacter* **(A,B)**, *E. coli* **(C,D)**, and *S. enterica* ssp. *enterica* **(E–G)**. Averages of the three independent replicate experiments of the controls without the product (0 μg/mL) and the exposures for 90 min to concentrations of BIOCITRO^®^ of half the lowest, the lowest, the highest and twice the highest MBC of each genus are shown.

**FIGURE 3:**
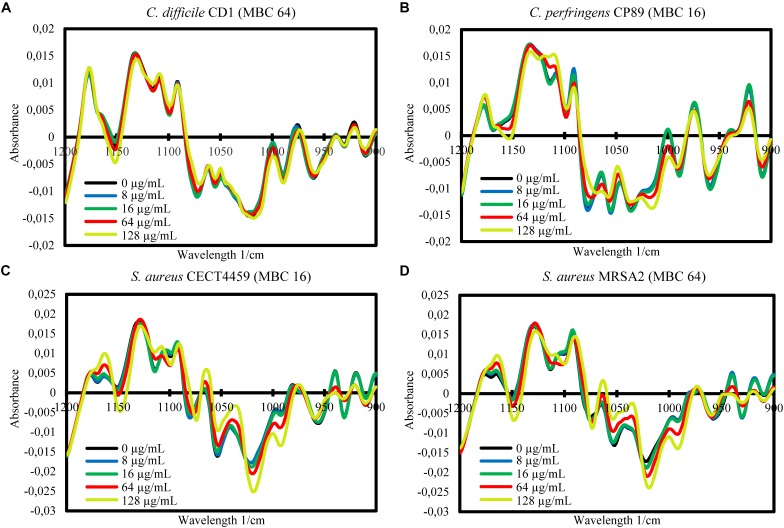
Second-derivative transformation of the FTIR absorbance spectra of the w_4_ window of the selected Gram-positive strains of *Clostridium* **(A,B)** and *S. aureus* **(C,D)**. Averages of the three independent replicate experiments of the controls without the product (0 μg/mL) and the exposures for 90 min to concentrations of BIOCITRO^®^ of half the lowest, the lowest, the highest, and twice the highest MBC of each genus are shown.

The FTIR spectroscopy showed variations between the spectra of the cells exposed to the product and the control cells in a wide range of the MIR spectral windows considered (w_1_ to w_5_). In order to study in more detail the differences between spectra, further mathematical analyses were carried out for the most reproducible and variable window, w_4_. For this window, the averages of the processed spectra of the three replicate experiments for each treatment and the control were represented for each strain (Figure 2, 3). Although after the exposure of the strains to BIOCITRO^®^ no changes in common wavelengths were observed for all species, most of the differences were found in the same regions at genus level. At the level of individual strains, a great range of wavelengths in the window showed small to large differences between the spectra of the cells exposed to the product and the control. In general, for both Gram-negative and Gram-positive strains, differences with regard to the control were observed from exposures to ½LMBCg at least in one region of the window with the exception of *C. perfringens* CP89 which showed differences from the LMBCg. However, most of the spectral regions showed no differences between the spectra of the control and those of the exposures to ½LMBCg or even to the LMBCg. Moreover, the slightest changes were displayed for *C. difficile* CD1 whereas it was difficult to assign the highest changes to a specific strain. Surprisingly, the species *S. enterica* ssp. *enterica* showed a mild decrease in the effect after the treatment with 2HMBCg respect to the exposure to the HMBCg.

**FIGURE 4:**
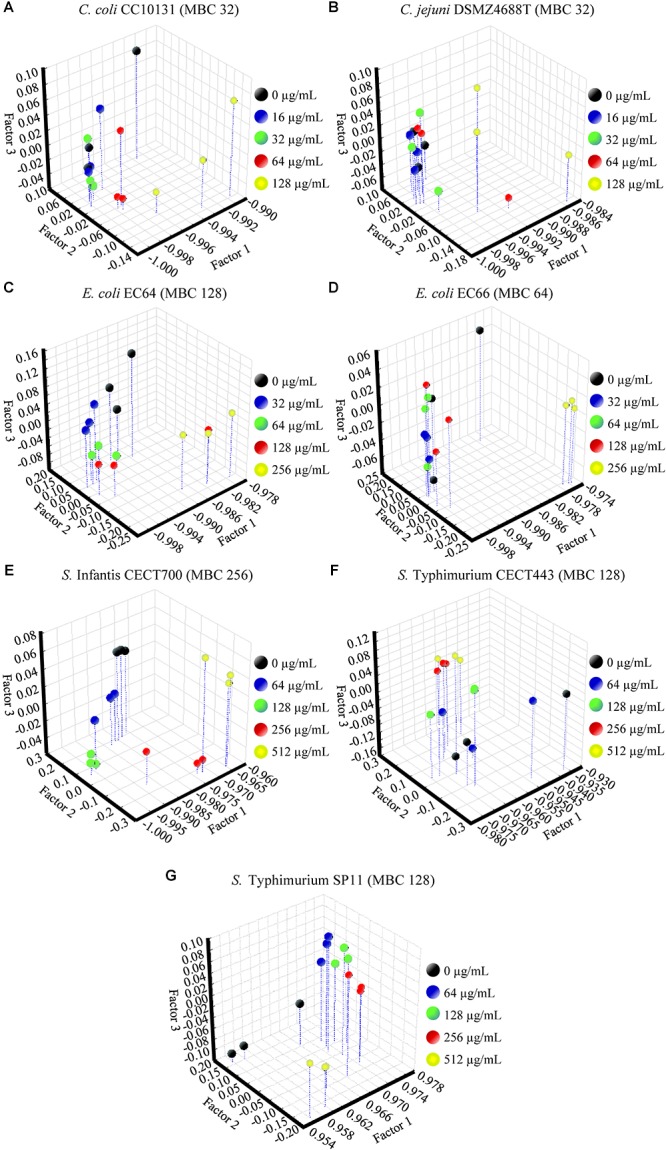
Factor analysis from ASCII data of the absorbance spectra of the FTIR w_4_ window of the Gram-negative strains of *Campylobacter* **(A,B)**, *E. coli* **(C,D)**, and *S. enterica* ssp. *enterica* **(E–G)**. Three independent replicate experiments of the controls without the product (0 μg/mL) and exposures for 90 min to different concentrations of BIOCITRO^®^ (half the lowest, the lowest, the highest, and twice the highest MBC of each genus) were included in the analysis.

**FIGURE 5:**
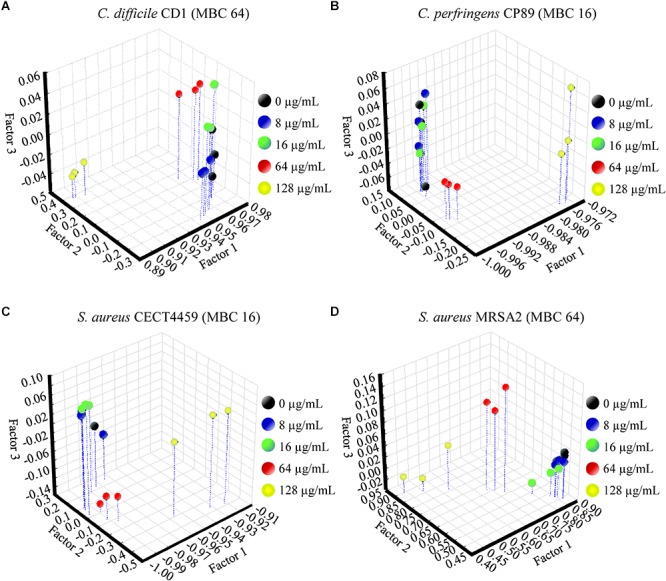
Factor analysis from ASCII data of the absorbance spectra of the FTIR w_4_ window of the Gram-positive strains of *Clostridium* **(A,B)** and *S. aureus* **(C,D)**. Three independent replicate experiments of the controls without the product (0 μg/mL) and exposures for 90 min to different concentrations of BIOCITRO^®^ (half the lowest, the lowest, the highest, and twice the highest MBC of each genus) were included in the analysis.

The FA of the three independent experiments for each strain showed the significance of the total differences between the spectra of the cells exposed to the product and the control cells for the w_4_ window (Figure 4, 5). A clear segregation was found in all the strains for the group exposed to 2HMBCg reflecting marked changes with regard to the controls. Nevertheless, clear segregations of practically all the spectra by treatment were found only in *E. coli* EC64, *S.* Infantis CECT700 and *S.* Typhimurium SP11 strains.

### SEM

**FIGURE 6:**
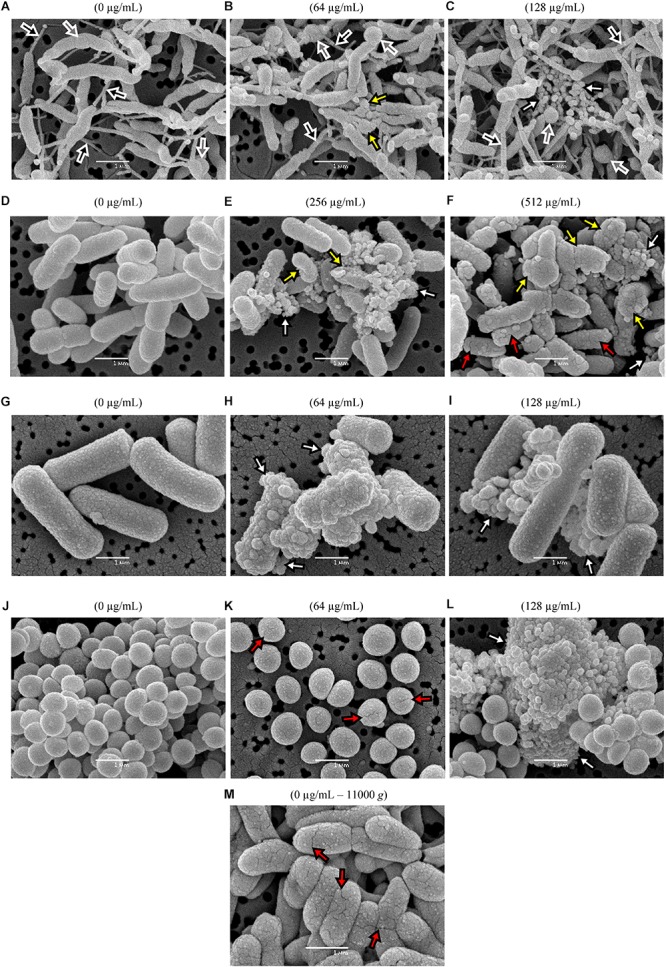
Qualitative SEM micrographs of two Gram-negative and two Gram-positive representative species after exposures for 90 min to different concentrations of BIOCITRO^®^ (highest and twice the highest MBC of each genus) compared with the controls without product (0 μg/mL). The strains of *C. jejuni* DSMZ4688T **(A–C)**, *S.* Typhimurium CECT443 **(D–F)**, *C. perfringens* 89 **(G–I)** and *S. aureus* CECT4459 **(J–L)** are shown. The fissures found in the control of the strain *S.* Typhimurium CECT443 after its centrifugation to high gravitational forces are shown in **(M)**. Depending on the color inside the arrows, the black arrows point to nanotubes, the gray arrows show swellings and free spherical bodies, the white arrows blebs and microvesicles, yellow arrows show collapsed and deformed cells and red arrows point to fissures.

With the aim of characterizing qualitatively the physical and morphological changes caused by BIOCITRO^®^, *C. jejuni* DSMZ4688T, *S.* Typhimurium CECT443 as Gram-negative representatives, and *C. perfringens* CP89 and *S. aureus* CECT4459 as Gram-positive representatives were selected. At first, following a protocol which recovered cells using centrifugation at 11000 *g*, fissures were found in the control of *S.* Typhimurium without the product (Figure 6M). After this observation, all the protocols used in this study were adapted by avoiding centrifugations before the treatments to minimize previous damage associated to the handling of the sample.

All the four strains showed drastic changes when they were exposed to their HMBCg and 2HMBCg, as shown in the representative micrographs selected for the Figure 6. *C. jejuni* DSMZ4688T displayed a nanotube network connecting practically all cells, even in the case of the control without product. Moreover, large spherical swellings were observed in some cells of this strain as well as similar spherical bodies as free forms. These swellings and spherical bodies increased from the control to the exposure to the highest concentration. This net of nanotubes, swellings and spherical bodies were specific of this strain and were not observed in the remaining species. A common effect in the four selected strains was the occurrence of microvesicles which were observed only after the exposure to BIOCITRO^®^, with a strong increase in 2HMBCg. In addition, blebs of similar size of the microvesicles were found in the surface of some of the treated cells, less apparent in the case of *C. jejuni*. Some of the cells of the selected Gram-negative strains showed the loss of their structural integrity and became deformed and collapsed even with the HMBCg, whereas cells observed from the Gram-positive strains kept their shape even with expositions to 2HMBCg. Another effect was the appearance of fissures on the surface of *S.* Typhimurium cells and, together with separated cells, in the case of *S. aureus*.

### Survival Test

**FIGURE 7:**
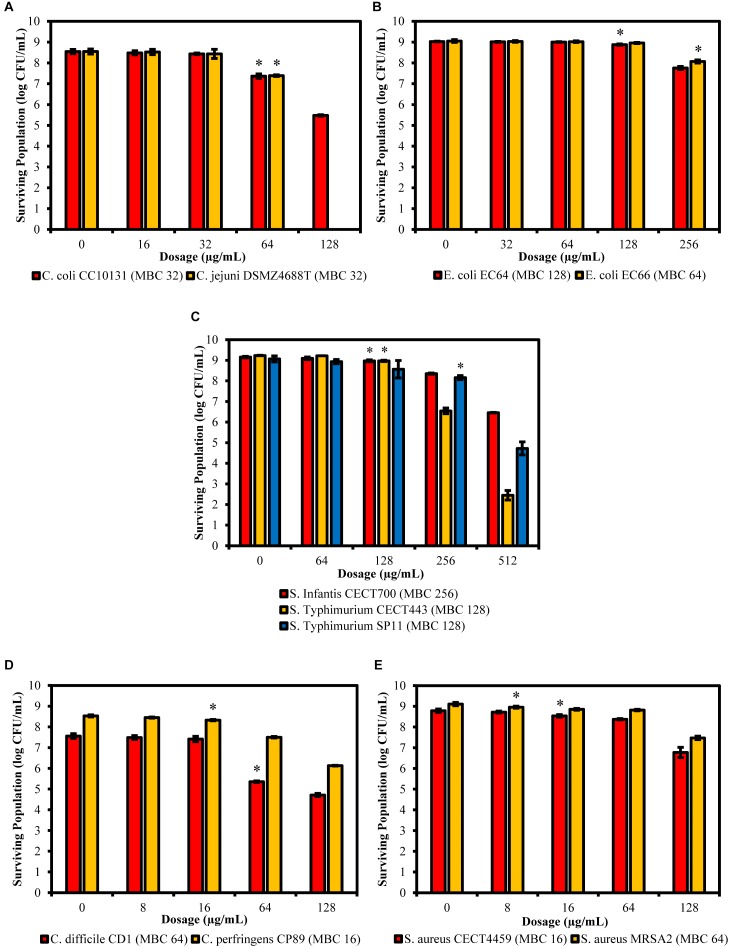
Surviving populations of the selected strains of *Campylobacter* **(A)**, *E. coli* **(B)**, *S.* Typhimurium **(C)**, *Clostridium* **(D)**, and *S. aureus* **(E)** after exposures for 90 min to concentrations of BIOCITRO^®^ of half the lowest, the lowest, the highest and twice the highest MBC of each genus. The differences between the average of damaged cells of each treatment with regard to the control (without product) were significant for the concentrations marked with ^∗^ and higher.

The viable cells of the controls and the cultures exposed to the product were estimated by counting the number of CFU after plating suitable dilutions of each treatment onto solid media. The results showed that the significant effect over the viability of the cells began at ½LMBCg only for *S. aureus* MRSA2, which was the only one that shows a significant decrease in cell viability with regard to the control (*P* = 0.047) at the lowest concentration of 8 μg/mL (Figure 7). On the contrary, *E. coli* EC66 and *S.* Typhimurium SP11 were the strains with the highest concentration (256 μg/mL) from which a significant reduction of cells was achieved (*P* ≤ 0.001). The significant decrease in cell viability for the remaining strains started at concentrations of 16 μg/mL for *C. perfringens* CP89 and *S. aureus* CECT4459, at 64 μg/mL for the genus *Campylobacter* and *C. difficile* CD1, and at 128 μg/mL for *E. coli* EC64, *S.* Infantis CECT700 and *S.* Typhimurium CECT443. Only the concentrations of 64 and 128 μg/mL were used for all strains and could be interspecifically compared; all strains showed a significant reduction of cells at 64 μg/mL with the exception of *E. coli* and *S. enterica*, which reached a significant reduction of cells with 128 and 256 μg/mL, depending on the strain. On the other hand, under this conditions of incubation of the stationary phase cultures, in five of the 11 strains the concentration at which a significant reduction in the number of cells was achieved, matched their MBC obtained using the microdilution method. Nevertheless, four strains differed in one dilution step with regard to the MBC, *E. coli* EC66 showed the significant reduction of cells at four times its MBC and for *S. aureus* MRSA2 this value was eight times lower than its MBC.

The maximum amount of non-viable cells with regard to the control was 8.55 log of CFU/mL for *C. jejuni* DSMZ4688T that showed no viable cells after its exposure to its 2HMBCg. However, the minimum amount of non-viable cells at 2HMBCg was found in *E. coli* EC66 with only 0.98 log of CFU/mL of the 9.05 log of starting CFU/mL. For the whole study, the average in the decrease of the viability of the cells was 3.33 log of CFU/mL of an average of the controls of 8.78 log of CFU/mL.

## Discussion

Using different techniques, we determined the bacteriostatic and bactericidal concentration of BIOCITRO^®^ and we elucidated the behavior of different bacteria when they are exposed to this product and the molecular changes which take place in their cellular envelopes. The assessment of the viability of the cells after the treatments provided relevant information to explain the effects observed using other techniques on the survival of the bacterial cells.

### MIC and MBC

BIOCITRO^®^ showed antimicrobial activity against all the strains of the pathogenic genera tested, as previously shown for *B. hyodysenteriae* and some food-borne pathogens (Bevilacqua et al., 2010; de Nova et al., 2017) as well as for other citrus extracts (Negi and Jayaprakasha, 2001; Mandalari et al., 2007; Alvarez-Ordóñez et al., 2013; Torres-Alvarez et al., 2017; Tsiraki et al., 2018). In most strains tested, the MBC was the same as the MIC, showing that the product is almost always bacteriostatic and bactericidal at the same concentration, as it was previously described for a similar citrus extract with slightly higher activity against three Gram-negative bacteria (Alvarez-Ordóñez et al., 2013). In general, its activity against Gram-positive bacteria was higher than that against Gram-negatives. Despite this, the highest MBC for the Gram-negative genera was 32 μg/mL for *Campylobacter*, 128 μg/mL for *E. coli* and *B. hyodysenteriae* (de Nova et al., 2017) and 256 for *S. enterica* ssp. *enterica*. The values of the 50 and 90% of inhibition (MIC) or death (MBC) of the cells of all strains within each bacterial group were different only for the MBC of *S. enterica* ssp. *enterica* and the MIC and MBC of *S. aureus*, showing the uniform behavior of most strains within each group.

The composition of this citrus extract (see text footnote 1) may explain its activity. It includes bioflavonoids as hesperidin, naringin, rutin, and quercetin, together with ascorbic acid, which have been previously described as compounds with antimicrobial activities (Rauha et al., 2000; Yi et al., 2008; Ozçelik et al., 2011). The potentiation of the antimicrobial effects of quercetin has also been described for rutin and ascorbic acid (Arima et al., 2002; Kallio et al., 2012). Although the composition is very similar to BIOLL^®^ (Alvarez-Ordóñez et al., 2013), this study demonstrated certain differences in susceptibility and the mode of action between them, showing different behaviors of the citrus extracts. The results of this study agree with the recommended dosage as feed additive for the liquid presentation of BIOCITRO^®^, which ranges from 100 to 300 μg/mL. Moreover, the observations after the use of the product in pig, ruminants and poultry production indicate that it reduces digestive disorders and helps in the control of *Clostridium* spp., *E. coli*, and *Salmonella* spp. infections. In addition, as it is considered raw material without any toxic effects and free of undesirable substances, there is no restriction or limit in the maximum dosage, making it possible to increase the amount for therapeutic purposes without any risks.

### Evaluation of the Mechanism of Action

In order to assess the mode of action of the product, we used stationary phase cultures instead of logarithmic ones taken into account several evidences. First of all, it must be considered that in an infection there may be no optimal growth conditions for the bacteria. Moreover, non-multiplying or slowly growing organisms have been shown as generally less susceptible to antibacterial agents (Eng et al., 1991). It has been also described that *E. coli* stationary phase cultures are adapted to resist adverse environmental conditions (Pletnev et al., 2015) and the exponential phase cells of *Pseudomonas aeruginosa* are more susceptible to the antimicrobial action of tea tree oil than stationary phase cells (Longbottom et al., 2004). For all these reasons, the use of cells in the logarithmic phase of growth may show higher activity of the evaluated product than cells in the stationary phase, making it advisable to use cells in the stationary phase of growth for evaluation of susceptibility. In addition, it has been described that PI could stain growing cells of some bacteria (Shi et al., 2007), making it more advisable to use cells in stationary phase in the case of the FC technique.

An increase in the number of cells with altered membrane permeability to PI related to the concentration of BIOCITRO^®^ was shown for most of the strains tested using FC. This effect was significant compared to the control of each strain at 16 μg/mL for the genus *Clostridium*, at 32 μg/mL for *C. coli* and at 64 μg/mL for the remaining species. Maximum detectable damage using this technique was reached after the exposure to 256 μg/mL of the product for the whole strains tested. This is consistent with the data obtained for *B. hyodysenteriae* in a previous study (de Nova et al., 2017) and is similar to the effect described for BIOLL^®^ using a similar approach (Alvarez-Ordóñez et al., 2013), with the exception of *Salmonella enterica* ssp. *enterica* strain SP11 which showed in this study an important decrease in the number of stained cells with regard to the observed for BIOLL^®^, demonstrating differences between these two commercial citrus extracts. On the other hand, the mild damage detected in the controls may be due to the ability of PI to stain growing cells (Shi et al., 2007) or in the case of *Clostridium* spp. to damages associated with the exposition to oxygen during the manipulation of the samples. It is important to denote that this technique is incapable of detecting the lysis of the cells as it can only count the cells which remain unbroken. Thus, the real effect of the product in the membrane integrity may be underestimated if a significant amount of cells is disintegrated by the product. This may be the case of the strain *C. jejuni* DSMZ4688T that the method could not detect cells with PI, due to a special behavior of its membrane. Conversely, PI inside the cells reflects the alteration of the membrane permeability but does not indicate that all the affected cells have lost their viability, so the antimicrobial effect of the extract can be overestimated. However, this technique was useful to prove that one of the effects of BIOCITRO^®^ takes place on the membrane permeability and in combination with the remaining techniques could elucidate the mode of action of the product against bacteria.

The activity of the citrus extract on the structure and composition of the cells was determined by the evaluation of the changes in the MIR spectra after the incubation for 90 min with and without BIOCITRO^®^. Differences between most of the spectra of each strain were observed in all the five spectral windows considered, suggesting a wide range of targets affected by BIOCITRO^®^ as it was described for BIOLL^®^ (Alvarez-Ordóñez et al., 2013). Nevertheless, in this study the most altered window was the w_4_, instead of the w_3_, which reflects certain differences in the mode of action of the two extracts. The window w_1_, called the fatty acid region, reflects changes in fatty acids. The amide region, the w_2_ window, displays variations in the amide I and II groups of proteins and peptides. The mixed region w_3_ detects the influence in proteins, fatty acids and compounds with phosphate groups. The most variable window of this study (w_4_), the polysaccharide region, mostly reflects alterations in polysaccharides and carbohydrates of the cell wall. Some authors also described that the w_4_ window may reflect alterations in the nucleic acids at ∼1160, ∼1120 and 1076 for their backbones and at 1085 for their phospholipids (Yu and Irudayaraj, 2005; Prudêncio et al., 2015). And finally, the w_5_, named the true fingerprint, conforms a species-specific spectral pattern (Dziuba et al., 2007; Alvarez-Ordóñez and Prieto, 2012; Prieto-Calvo et al., 2014). According to this, the main effect of BIOCITRO^®^ was focused on carbohydrates, polysaccharides and nucleic acids, although a mild effect over fatty acids and proteins was also observed.

Although there were similar spectra at genus level for the w_4_ window, the strong differences between genera in this window did not make it possible to associate a general pattern of changes for all genera to specific wavelengths. In most cases, the changes were related to the concentrations used, with the special case of some wavelengths for *Salmonella enterica* ssp. *enterica*, which showed a low decrease of the effect after their exposure to 2HMBCg with regard to the exposure to the HMBCg. This fact may reflect a threshold above which it is no longer possible to detect using this technique the effect on some altered compounds or a loss of information due to the lysis of some cells. Moreover, the changes were noticeable at least for one wavelength starting with exposures to ½LMBCg, although control samples generally showed the same spectra as exposures to ½LMBCg or even the LMBCg for most wavelengths. This behavior was shown in the segregation of the groups of the different exposures to the product using FA, where most of the strains showed no differentiation between exposures to the two lowest concentrations and the control. On the other hand, only three strains (*E. coli* EC64, *S.* Infantis CECT700 and *S.* Typhimurium SP11) showed an acceptable definition of their five groups of expositions but it was not possible to associate this behavior to a genus or a bacterial envelope. Nonetheless, only three strains showed no more than one different group of the untreated cells.

Severe morphological alterations were found in all micrographs obtained qualitatively using SEM after the exposure of the selected strains to BIOCITRO^®^. Common effects over all the strains were observed as blebs on the surface of the cells and microvesicles as free forms. The morphological similarity between these two structures suggests the possibility that blebs were the source of the microvesicles detected as free forms. Blebs seem to be a common bacteria response to antimicrobials, as previously described for *S. enterica* (Kumaresan et al., 2015), *S. aureus* (Carvajal-Rondanelli et al., 2018) and other Gram-negative and Gram-positive bacteria (Takenouchi and Nishino, 1991; Tagai et al., 2011; Ryu et al., 2014; Prudêncio et al., 2015; Zhang et al., 2016). On the other hand, in this study only the Gram-negative species collapsed when exposed to the product, suggesting a different response of the two kinds of bacterial envelopes to BIOCITRO^®^. Collapse of the cells were previously described after the exposure of the cells to some antimicrobials for both the Gram-negative *E. coli* (Wang et al., 2007; Zhou et al., 2010; Jońca et al., 2016) and the Gram-positive *S. aureus* (Hu et al., 2011). In addition, a remarkable effect over the arrangement of the cells of *S. aureus* was observed, turning from grape-like clusters to individualized cells as it was previously observed when they were exposed to other plant extracts (Hu et al., 2011). Moreover, a large number of fissured cells was evidenced among the individualized cells, indicating that the disintegration of the normal grape-like clusters occurs together with a greater fragility of the cells. Fissures were observed only after the exposure of *S.* Typhimurium and *S. aureus* to the product, but only in collapsed cells for the first one and in the individual cells for the last one. This fact suggests that the appearance of fissures is a consequence of the fragility of the cells due to the treatment even when the cell shape is maintained as in the case of *S. aureus*. A possible greater elasticity of the *C. jejuni* envelope and the absence of collapsed cells for *C. perfringens* may explain the absence of fissured cells for these two species.

In a similar way, the spherical swellings in the case of *C. jejuni* seem to be the precursors of the spherical bodies. The change from spiral to coccoid forms after exposition to magnesium oxide nanoparticles has been described for this species (He et al., 2016), supporting the formation of coccoid forms in harsh environments. Besides, the presence of nanotubes in the strain of *C. jejuni* is not a common observation in liquid medium cultures. BIOCITRO^®^ previously showed an increase in the formation of nanotubes by *B. hyodysenteriae* (de Nova et al., 2017), but in the case of *C. jejuni* DSMZ4688T their presence could not be associated with the exposure to the citrus extract since it also reached a high density in the control. Nanotubes are described to be involved in the exchange of cytoplasmic molecules within and between species, including trade between Gram-positive and Gram-negative species (Dubey and Ben-Yehuda, 2011; Baidya et al., 2018) even when the cells lack nutrients (Pande et al., 2015). The presence of nanotubes and swellings in *C. jejuni* control cells possibly reflects the suboptimal culture conditions to which this species was subjected from the beginning, but differences between treated cells and controls were clear and informative of the effects of the extract.

It is worth noting that the discovery of the damage produced in the bacterial cells when they were subjected to very high gravitational forces makes the verification of the cellular integrity using SEM advisable. Centrifugation of the sample should be avoided when possible, and the manipulation of the sample reduced to a minimum. On the other hand, the observation using SEM of the morphological and physical changes that the cells undergo after their exposure to the product is very important to understand the behavior of the cells that produces the alterations at molecular level displayed using the other techniques.

After the assessment of the changes produced by BIOCITRO^®^ in the selected bacteria using FC, FTIR spectroscopy and SEM, it was evident that this product induced drastic changes in the permeability, structure, composition and morphology of the bacterial envelope. These changes reflected its mode of action but do not necessarily compromise the viability of all the affected cells. The survival test showed the real impact of the changes observed using the other techniques after the exposure to the product on the viability of the cells. It resulted crucial to elucidate whether the bacteria remain viable even with evident damages. According to our results, it should be mandatory (or highly recommended) in all assessments of the mode of action of antimicrobials to avoid that reversible damages in the bacteria being considered as a decrease in the viability of the cells, and therefore, as a false increase in the susceptibility to the product. In addition, it is important to remark that the use of graphs of surviving population in the assessment of the survival test or in other studies of inactivation kinetics should be preferred instead of graphs of reduction in the number of cells which do not give information about the initial viable cell concentration of the culture. A reduction of 8 log of CFU/mL in an initial culture of 8 log of CFU/mL corresponds to a 100% reduction, while it is only a 10% decrease if the initial concentration is 9 log of CFU/mL. Moreover, the absence of information on the initial concentration of cells does not allow to clarify whether the differences in susceptibility between strains are due to the exposure of different amounts of cells to the product. If the product is inactivated after its action over the cell, small amounts of cells should exhibit greater susceptibility than large amounts of cells of the same strain exposed to the same concentration of the product.

This study showed a high variability in the viability of the bacteria after their exposure to BIOCITRO^®^ for 90 min. Overall, from an average of 8.78 log of starting CFU/mL, an average of 3.33 log of reduction of CFU/mL (range 0.98–8.55) was achieved when the strains were exposed to their 2HMBCg. Moreover, while some strains as *E. coli* EC66 and *S.* Typhimurium SP11 were not significantly affected after their exposure to 128 μg/mL of the product, the strain *C. jejuni* DSMZ4688T showed no viable cells at this concentration and it was the only strain that reached the maximum damage at these conditions. The minimum concentrations at which a significant decrease in the viability was reached were also highly variable (from 8 to 256 μg/mL), showing Gram-positive strains as having the highest susceptibility (between 8 and 64 μg/mL). On the contrary, *E. coli* and *S. enterica* ssp. *enterica* were the strains with the lowest susceptibility and reduction of viable cells was lower than previously described for these bacteria after exposure to similar concentrations of a comparable citrus extract (Alvarez-Ordóñez et al., 2013). However, the method used in that study included centrifugation of the cells of the stationary phase cultures before the treatment at high gravitational forces (11000 *g*), a fact which could produce fissures in the cells, as was demonstrated using SEM in our research, and may increase the susceptibility of the bacteria to the product. On the other hand, it was observed that the lowest concentrations at which significant reduction of cells were achieved after 90 min of exposition were the same or differed in one dilution step of the MBC of the strains with the exception of *E. coli* EC66 and *S. aureus* MRSA2. This suggests that the lowest concentration at which significant reduction of cells in the stationary phase was detected is approximately the same concentration which kills the bacteria after the culture conditions for MBC for most of the strains. However, differences found in two of the tested strains indicate that results of survival test should not always correlate with biological relevance.

Furthermore, the survival test showed the real significance of the effect over the membrane permeability displayed using FC. Most of the strains showed higher percentages of inactivated cells than PI stained cells at all the concentrations tested suggesting that probably lysis or collapse of some of the cells occurs. On the contrary, a higher percentage of PI stained cells was observed for *E. coli*, *S.* Infantis CECT700 and *S.* Typhimurium CECT446 strains indicating that some of the cells were viable in spite of alterations of their membrane permeability.

In addition, the fact that *C. jejuni* showed no viable cells after its exposure to twice the maximum MBCg indicates that the cells observed using SEM at this concentration were not viable despite maintaining their shape. This supports that the microvesicles observed as free forms may be not the result of the cell lysis but of their release by the cells in response to an envelope stress as it has been previously described for Gram-negative bacteria (McBroom and Kuehn, 2007).

With regard to the behavior of the phagotype DT104 of *S.* Typhimurium, which was included due to its particular resistance to antibiotics, the number of non-viable cells was higher than the number of cells with PI at all concentrations evaluated in contrast to the other strains of *S. enterica*. Despite this, no differences in susceptibility were identified, displaying similar MBC and a decrease in viable cells than the other *S. enterica* strains evaluated.

## Conclusion

This study confirms the *in vitro* antibacterial activity of BIOCITRO^®^ against Gram-negative and Gram-positive bacteria. For most of the strains, the product reached the bactericidal effect at the same concentration of the bacteriostatic effect and maximum difference between MIC and MBC was two dilution steps. The less susceptible species of the study were *S. enterica* ssp. *enterica* and *E. coli* with MBC_90_ values of 256 and 128 μg/mL, respectively, while the most susceptible was *C. perfringens* with MBC_90_ of 16 μg/mL. After short exposition time to the product, the significant effect over the viability of the stationary phase cells ranged from 8 to 256 μg/mL. With regard to the mode of action of the product, by means of FC, FTIR spectroscopy and SEM we demonstrated that it causes changes in the permeability, structure, composition and morphology of the bacterial envelope in a similar way to other citrus fruit extracts. Changes affected practically all components of the cell envelope but mainly to carbohydrates and polysaccharides of the cell wall. Furthermore, the alteration of the membrane permeability increased with the concentration of the product, although it did not always affect the viability of the cell, as was observed for the strains of *E. coli* and two of the strains of *S. enterica* ssp. *enterica*, which displayed using FC higher number of cells with altered membranes than non-viable cells at the same concentration. In addition to other specific effects, there was a general response of the cells releasing microvesicles when they were exposed to the product. Besides, it was observed using a survival test that some of the cells observed using SEM may be inactivated even maintaining their shape. Moreover, the susceptibility to BIOCITRO^®^ of the antibiotic multiresistant strain *S. aureus* MRSA2 and the phagotype DT104 of *S.* Typhimurium allow us to suggest that the product may have a different mode of action of the antibiotics.

On the other hand, the use of very different techniques to assess the mode of action of the product showed that the information given using each single technique may be misunderstood if it is not compared with other techniques, especially with the survival test that clarify the real effect of the changes detected using other techniques over the viability of the cells. According to this, we suggest that this technique should be mandatory in studies of the mode of action of antibacterial products. SEM also provided relevant information on the integrity of the cells after the manipulation of the samples. Therefore, the verification of the cellular integrity using SEM in the development of any technique that uses cell cultures should also be highly recommended to ensure that the manipulation of the sample does not affect the quality of the data obtained.

## Author Contributions

PN, AC, MP, and PR conceived and designed the experiments, analyzed the data, and wrote the manuscript. PN performed the experiments.

## Conflict of Interest Statement

The authors declare that the research was conducted in the absence of any commercial or financial relationships that could be construed as a potential conflict of interest.

## Footnotes

1 http://probenasl.com/?page_id=1157\&lang=en

2 http://www.who.int/
